# A review of management options for splenic artery aneurysms and pseudoaneurysms

**DOI:** 10.1016/j.amsu.2020.08.048

**Published:** 2020-09-09

**Authors:** Hse Juinn Lim

**Affiliations:** Department of General Surgery, Royal Gwent Hospital, Newport, UK

**Keywords:** Splenic artery aneurysms, Vascular surgery, General surgery, Upper GI surgery, Emergency medicine

## Abstract

**Background:**

A review of the management of splenic artery aneurysms (SAA). There is no general consensus as to when and what type of intervention should be chosen to treat SAAs. The aim of this study is to investigate the types of intervention for SAAs including complication, reintervention, rupture, mortality after intervention of SAA in a review.

**Method:**

A literature search was performed using “keywords” in Medline and Embase limited to publications from 2008 to 2018. 289 articles were identified during the initial literature search. 143 articles met the eligibility criteria. 83 articles were included in the quantitative synthesis. Descriptive analysis was performed.

**Results:**

576 patients were identified with 588 reported SAAs. The mean ± SD age was 52.6 ± 5.8 years (range 17–85). The mean ± SD size of SAA was 49.9 ± 13.2 mm (range 6–180). Types of intervention reported were endovascular treatment, open surgery, laparoscopic surgery and conservative management. Mortality rate in patients with endovascular treatment was 0.5% compared to 4.9% with open surgery. 3.4% of patients with conservative management were reported to have aneurysms that grew over time and 2.8% patients had further intervention. ANOVA test to compare mortality between open surgery, endovascular treatment and laparoscopic surgery showed there is no difference between mortality between the 3 different interventions as F (2.71) < F crit (3.02) (P = 0.07).

**Conclusion:**

Endovascular treatment is now the first choice of treatment for SAA, but future studies are required to determine its long-term durability. By introducing a management pathway for SAA, we hope to see an improvement in managing patients. The management algorithm will require further validation through application with careful and complete follow-up of all cases to improve the pathway depending on patient outcome.

## Highlights

• No general consensus type of intervention for splenic artery aneurysms.
• Endovascular surgery has lowest mortality rate compared to open and laparoscopic surgery.
• Endovascular is preferable choice but long-term durability needs to be determined.
• Choice of intervention depend on confidence of surgeon skills and patient preference.
• Management algorithm created to simplify choice of management using flowchart.

## 1 Introduction

Splenic artery aneurysm (SAA) is defined as an abnormal dilatation of splenic artery more than 1 cm in diameter [1]. It was first reported in 1770 by Beaussier and was described in a living person by Winkler in 1903 [[2], [3], [4], [5], [6], [7], [8], [9]]. SAA is an uncommon disease in the general population with incidence ranging from 0.09% in autopsy studies and 0.78% in arteriography studies [3]. Although rare, SAA is the third most common type of intraabdominal aneurysm and accounts for 40–60% of all cases of visceral artery aneurysms [1,4]. SAAs can be classified histopathologically into true aneurysms and pseudoaneurysms, which the later having a more catastrophic course than true aneurysms [1]. As the use of axial imaging techniques is increasingly used, the incidental detection of SAA is rising with 80% of asymptomatic SAA found incidentally [10]. The risk of rupture of SAAs ranges from 3% to 25% [5]. Mortality rate for ruptured SAAs is between 25% and 70%, especially if presented in patients with significant comorbidities or during pregnancy [5]. The first reported SAA operation was done on 1932 by Lindboe [4,7]. Since then many different treatment options for SAA has been reported in literatures such as conservative management, endovascular treatment, laparoscopic surgery and open surgery [4]. The aim of this review is to provide an overview of the management options for splenic artery aneurysms.

## 2 Material and methods

A literature search was performed using “keywords” in Medline and Embase limited to publications from 2008 to 2018. Keywords searches of splenic artery, aneurysm, pseudoaneurysm, aged, adult, middle-aged, young adult, elderly, conservative treatment, surgery, interventional radiology, treatment outcome, rupture, mortality, reintervention, follow up were used along with Boolean operators.

Case reports, case series, letters to editors, conference papers, review articles and original studies of SAAs were reviewed, and their reference lists evaluated. The language of the publication was limited to English only. Studies without the full text, an abstract with insufficient data, or studies with poor content for comparison were excluded. As the literature is depleted in the subject, articles on visceral artery aneurysms and patients with mixed pathologies are included only if adequate clinical information about patients was reported. Countries included China, Qatar, USA, Greece, UK, Japan, Italy, India, Canada, Slovak Republic, Turkey, Serbia, Kuwait, Iran, Brazil, Bosnia and Herzegovina, Taiwan, Poland, Australia, Ireland, Morocco, Croatia, Spain, Korea, Germany, Sri Lanka and France. Full texts were obtained using Cardiff University Library Search. Items not found on Cardiff University's library catalogue were loaned using Inter-library Loan Request Service.

289 articles were identified during the initial literature search. 86 articles were excluded during abstract/title screening. Of the remaining 203 articles, 20 duplicates were removed, and 27 full texts weren't accessible. 4 articles were identified through other resources. 143 articles met the eligibility criteria and 83 articles were included in the quantitative synthesis.

All statistical analysis and charting were performed with Microsoft Excel 2016 for Mac. No inferential statistical analysis was performed due to heterogeneity of patient population and treatment strategies. Only descriptive analyses are presented and discussed.

## 3 Results

**Fig. 1:**
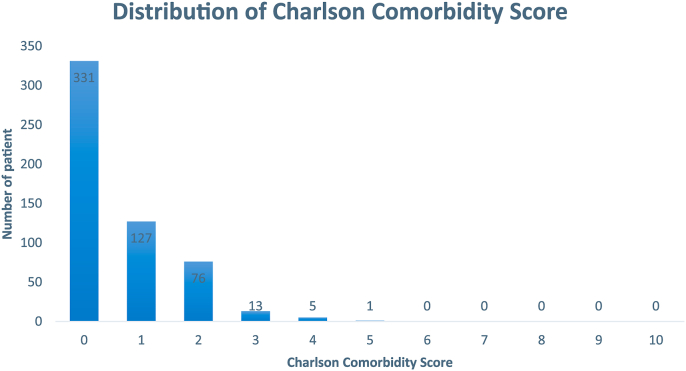
Histogram showing distribution of Charlson Comorbidity Score across 576 patients.

The literature review included 83 papers involving 576 patients with 588 reported splenic artery aneurysms. Of the 83 papers, 17 were original papers, 11 were case reports, 42 were case reports with literature, 3 were letter to editors, 7 were case reports with images, 1 was a technical note and 2 were conference papers. Out of the 588 aneurysms, 555 (94.4%) were true aneurysms and 33 (5.6%) were pseudoaneurysms. The location of 100 (17%) splenic artery aneurysms and pseudoaneurysms were reported, 30 (30%) proximal SAAs, 25 (25%) middle SAAs, 27 (27%) distal SAAs and 18 (18%) hilar SAAs. The mean ± SD age was 52.6 ± 5.8 years (range 17–85). Of the 576 patients, 227 (39.4%) were men and 348 (60.4%) were women. The female-to-male ratio is 1.53:1. Of the 588 SAAs, aneurysm dimensions were obtained for 572 (97.3%) SAAs. The mean ± SD size of SAA was 49.9 ± 13.2 mm (range 6–180). 45 (7.7%) ruptured aneurysms were recorded including both pre-intervention and post-intervention. Patients’ comorbidities data was available for Charlson Comorbidity Index (CCI) (Fig. 1).

**Fig. 2:**
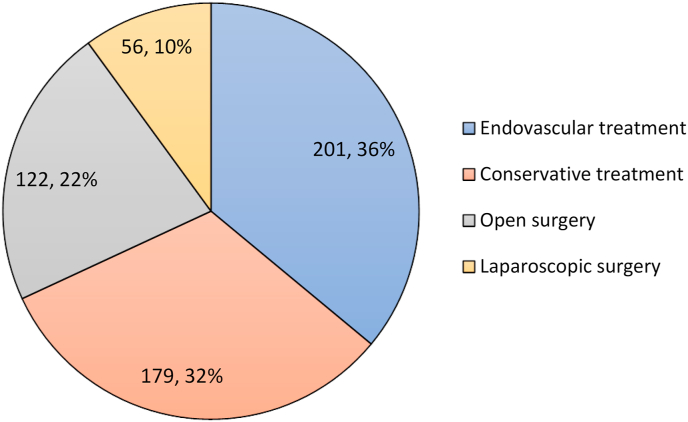
Number of patients with intervention for splenic artery aneurysm and pseudoaneurysm.

The median CCI obtained in individual patients was 0 (min = 0, max = 5). Out of the 576 patients with splenic artery aneurysms and pseudoaneurysms, the management of 558 patients was reported. (Fig. 2).

122 (21.9%) patients were managed with open surgery. There was 6 (4.9%) mortality in patients who had open surgery. 12 (9.8%) patients had post-surgery complications and 5 (4.1%) patients required reintervention. The time of patient follow-up was reported in 30 patients with a mean ± SD of 19.2 ± 9.9 months (range 1–112). 2 patients who required reintervention was managed with endovascular coil embolization. Complications that patients had were pleuritic and abdominal effusion in 3 (2.5%) patients, haemorrhage in 2 (1.6%) patients, deep tissue wound infection in 1 (0.8%) patient, pancreatic fistula in 1 (0.8%) patient and ruptured SAAs in 2 (1.6%) patients.

201 (36.0%) patients were managed with endovascular treatment. There was 1 (0.5%) reported patient mortality during endovascular treatment. 50 (25%) patients had post-procedure complications and 11 (5.5%) patients required reintervention after treatment. Patient follow-up was reported in 100 patients with a mean ± SD of 14.9 ± 6.9 months (range 3 weeks–117 months). The type of endovascular treatment was recorded in 67 patients. 44 (65.7%) patients had transcatheter embolization of SAA done with a total of 46 embolization performed. 41 (89.1%) coil embolization, 3 (6.5%) glue embolization and 2 (4.3%) vascular plug embolization was performed. 23 (34.3%) patients had endovascular stent-grafts of SAA done. Location for endovascular access for each patient was recorded, with 11 (47.8%) transfemoral, 2 (8.7%) transaxillary, 4 (17.4%) transbrachial, 2 (8.7%) transhumeral and transbrachial, 1 (4.3%) transhumeral and 3 (13.0%) not specified.

56 (10.0%) patients were managed with laparoscopic surgery. There was no reported mortality in patients who had laparoscopic surgery. 3 (5.4%) patients had post-surgery complications, 1 (1.8%) patient had portal vein thrombosis and 2 (3.6%) had ruptured SAAs. No patients required reintervention. Patients were followed up with a mean ± SD of 23.3 ± 8.7 months (range 2 weeks–110 months). 9 (16.1%) patients were treated using robot-assisted laparoscopic surgery.

179 (32.1%) patients were managed with conservative management. Out of the 179 patients, 6 (3.4%) were reported to have aneurysms that grew over time and 5 (2.8%) patients had intervention to treat the SAA. Mean ± SD of patient surveillance time was 26.7 ± 3.2 months (range 2 weeks–50 months). No mortality or complication was reported in patients who had conservative management.

Single Factor Analysis of Factor (ANOVA) test is used to compare the mortality of open surgery, endovascular treatment and laparoscopic surgery. The results are displayed in table below. As F < F crit (P = 0.07), we can accept the null hypothesis that there is no difference between mortality between the 3 different interventions. (Table 1).

**Table 1:** Single Factor Analysis of Factor (ANOVA) test of mortality between patients undergoing open surgery, endovascular treatment and laparoscopic surgery.

SUMMARY									
*Groups*	*Count*		*Sum*			*Average*		*Variance*	
Open	122		4			0.032786885		0.031973987	
Endovascular	201		1			0.004975124		0.004975124	
Laparoscopic	56		0			0		0	
ANOVA									
*Source of variation*	*SS*	*df*		*MS*	*F*		*P-value*		*F crit*
Between groups	0.070159605	2		0.035079802	2.711829426		0.067714365		3.019727674
Within groups	4.863877335	376		0.012935844					
Total	4.934036939	378							

## 4 Discussion

The pathogenesis of SAAs is not fully understood [8]. It is observed to occur in majority of patients with hypertension, hepatitis B or C virus, chronic or acute pancreatitis, portal hypertension, cholelithiasis, liver cirrhosis, trauma, diabetes, segmental arterial mediolysis, pregnancy and atherosclerosis. Ologun et al. mentioned that the pathogenesis of SAAs includes hypertension, hormonal factors (associated with degeneration of internal elastic lamina and elastin formation), hemodynamic changes (increased blood volume, cardiac output and portal congestion), and medial degeneration. Changes histologically include atherosclerotic changes, artery dysplasia, fibromuscular dysplasia, calcifications, cystic medial degeneration, and intimal hyperplasia [11]. Sadat et al. describes that in pregnancy, hormones (oestrogen, progesterone and relaxin) and psychological changes affect the arterial wall, causing medial degeneration and stress on arterial wall that leads to aneurysmal dilatation [12,13]. In patients with portal hypertension, hormones like aldosterone and renin have been suggested to cause thinning of the arterial wall [13].

The management of SAA has always been decided based on the choice of the doctor/surgeon performing the intervention and the patient's decision with no general consensus to follow [4,14]. Traditionally, the threshold for repair of asymptomatic SAA has always been > 20 mm [15]. There are some literatures that have suggested a few guidelines on the management of SAA. Corey et al. suggested guidelines on the management of asymptomatic SAA, recommending repairing all SAA for young women who are pregnant or are planning to be pregnant, liver transplant recipients, SAA >25 mm in patients who are fit for an operation and all pseudoaneurysms [15]. The article also suggested that most lesions can be managed using endovascular treatment as it is a less invasive intervention with lower mortality rate [10,15]. Guidelines for surveillance of asymptomatic SAA was also recommended by Corey at el for lesions ≤ 25 mm with axial imaging every 3 years to monitor the growth of the aneurysm [15]. Goldberg et al. also recommended using a multidisciplinary approach and treating all splenic artery pseudoaneurysms regardless of the size at presentation due to high risk of rupture and mortality [16].

The type of intervention for SAA is decided after careful consideration by the surgeon taking into account age, sex, aneurysm location, dimension, complications, adequacy of collateral flow to liver and severity of clinical findings [15]. Endovascular treatment is now more commonly used as it is a low morbidity procedure performed under local anesthesia, allows a short hospital length of stay due to rapid recovery and improvement of digital subtraction angiography (DSA) technology and equipment [10,[17], [18], [19], [20]]. On the other hand, open surgery carries a risk of mortality which is reported to be 1%–3%, and also has a high perioperative complication rate of 9%–25% [10,12]. A combination of several different techniques may be necessary for some patients for example in patients who had initial embolization followed by open surgery or laparoscopic surgery [21]. However, based on the ANOVA test, we can conclude that there is no difference in mortality between open surgery, endovascular treatment and laparoscopic surgery.

**Fig. 3:**
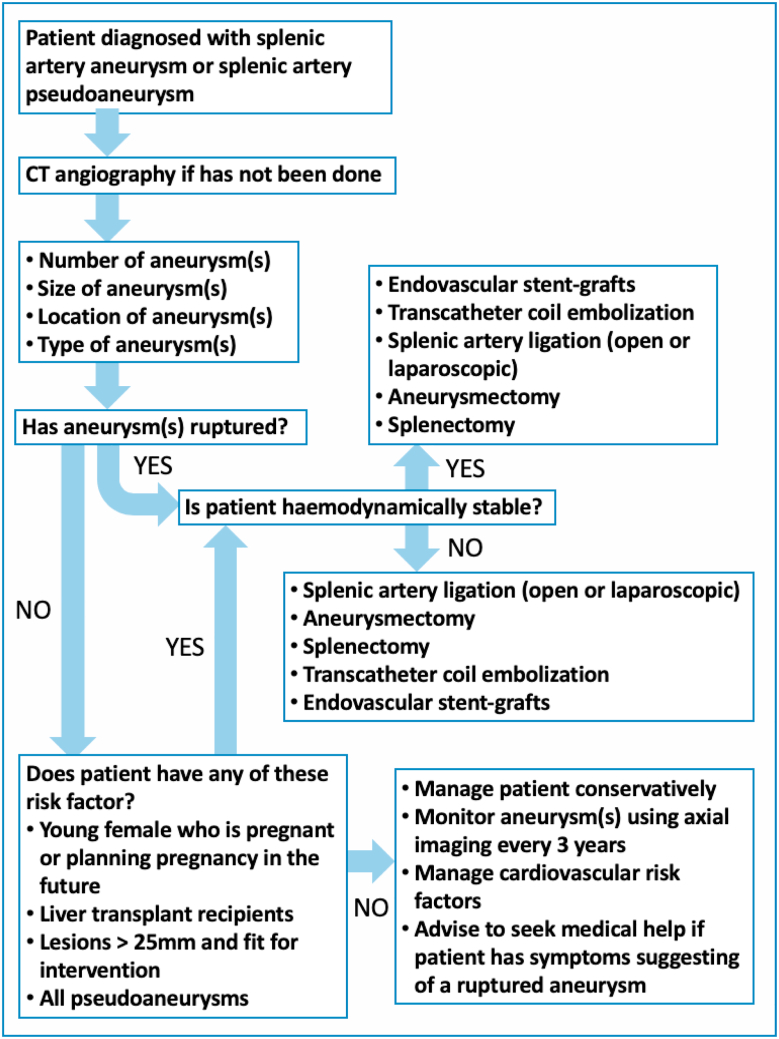
Management flowchart for splenic artery aneurysm or pseudoaneurysm.

A management flowchart has been synthesized to help in the process of determining the type of intervention for patients diagnosed with SAA or SAPA. (Fig. 3).

I would like to suggest an all UK registry of all diagnosed SAA and SAPA with a lifelong follow up to study and compare patient outcome of different. Based on the study we can then synthesize a guideline for management of SAA and SAPA.

## 5 Conclusion

This systematic review looked into the management of SAAs and the type of interventions for SAAs. Retrospective studies of SAAs are limited due to the rarity of SAAs leading to a small sample size. Conservative management needs to follow patient up until SAA ruptures, leaks or death of patient. Further studies are required to determine the long-term durability of endovascular treatment. Endovascular treatment is the standard of care for splenic artery aneurysms. However, the choice of intervention will be decided with careful discussion between patient and surgeon depending on confidence of surgeon skills and patient preference. The management flowchart will be applied to SAA cases in Aneurin Bevan University Health Board. By introducing a management pathway for SAA, we hope to see an improvement in managing patients with SAA. The management algorithm will require further validation through application with careful and complete follow-up of all cases to improve the pathway depending on patient outcome.

## Ethical approval

Not required.

## Source of funding

No funding was provided when conducting the review.

## Author contribution

Dr Hse Juinn Lim designed the project, collected data, analysed and interpreted data and wrote the data.

## Trial registry number

Name of the registry: Not required.

## Guarantor

Dr Hse Juinn Lim.

## Provenance and peer review

Not commissioned, externally peer reviewed.

## Declaration of competing interest

None.
